# Intravesical Allogeneic Platelet Concentrate for Refractory BK Virus-Associated Haemorrhagic Cystitis After Allogeneic Haematopoietic Stem Cell Transplantation

**DOI:** 10.3390/jcm15197407

**Published:** 2026-09-24

**Authors:** Ahmet Semih Güleser, Hasan Sami Göksoy, Zeynep Doğuşan, Dilek Ece, Atakan Eren, Aslıhan Sezgin, Nurgül Özgür Yurttaş, Özkan Onuk, Barış Malbora, Fatih Altunrende

**Affiliations:** 1 Department of Urology, Gaziosmanpaşa Hospital, Istanbul Yeni Yüzyıl University, 34010 Istanbul, Türkiye; 2 Department of Haematology, Gaziosmanpaşa Hospital, Istanbul Yeni Yüzyıl University, 34010 Istanbul, Türkiye; 3 Bone Marrow Transplantation Centre and Cell Processing Unit, Gaziosmanpaşa Hospital, Istanbul Yeni Yüzyıl University, 34010 Istanbul, Türkiye; 4 Department of Paediatric Haematology and Oncology, Gaziosmanpaşa Hospital, Istanbul Yeni Yüzyıl University, 34010 Istanbul, Türkiye; 5 Department of Urology, Istanbul Bilim University, 34394 Istanbul, Türkiye

**Keywords:** BK polyomavirus, haemorrhagic cystitis, allogeneic platelet concentrate, apheresis platelets, intravesical therapy, haematopoietic stem cell transplantation

## Abstract

**Background/Objectives:** BK virus-associated haemorrhagic cystitis (BKV-HC) is a severe complication of allogeneic haematopoietic stem cell transplantation (allo-HSCT) for which no standard treatment exists. Intravesical platelet-rich plasma has been proposed, but recipients are profoundly thrombocytopenic, and an autologous product cannot be obtained. We report the outcome of an intravesical allogeneic platelet concentrate (allo-PC) prepared from irradiated single-donor apheresis platelets in patients refractory to all available treatment. **Methods:** Single-centre retrospective series of 14 consecutive patients with Grade 3–4 BKV-HC treated between January 2018 and June 2021, selected from 84 patients who developed haemorrhagic cystitis during this period. All had failed hyperhydration, forced diuresis, continuous bladder irrigation and cidofovir; 10 had undergone previous cystoscopic cauterisation, 10 had immunosuppression reduction, and 4 had hyperbaric oxygen, all without resolution. The median duration of haematuria before allo-PC was 45.5 days. The primary outcome was resolution of macroscopic haematuria; transfusion requirement, catheter removal and readmission were reported separately. **Results:** Macroscopic haematuria resolved in 12 of 14 patients (85.7%, 95% CI 60.1–96.0) after a median of 5.5 days (IQR 4.0–12.5). Seven patients (50.0%, 95% CI 26.8–73.2) met all secondary criteria. Product platelet concentration correlated inversely with time to resolution (r_s_ = −0.917, 95% CI −0.977 to −0.725). Because a fixed 50 mL volume was given, the delivered dose varied 18-fold (0.30–5.62 × 10^9^ platelets/kg). Body weight also correlated with time to resolution (r_s_ = +0.791), but in partial correlation the association with platelet concentration persisted after adjustment for weight (r_s_ = −0.783, *p* = 0.004) and for age (r_s_ = −0.827, *p* = 0.002), whereas the association with weight did not persist after adjustment for platelet concentration (r_s_ = +0.292, *p* = 0.38). No procedure-related complication exceeded Clavien–Dindo Grade I. **Conclusions:** In patients who had exhausted all available treatment, intravesical allo-PC was followed by resolution of haematuria in most cases. The association between product platelet content and time to resolution was not explained by body weight or age. These uncontrolled findings are hypothesis-generating.

## 1. Introduction

Haemorrhagic cystitis (HC) is a clinically significant complication of allogeneic haematopoietic stem cell transplantation (allo-HSCT) [1]. Early-onset cases arising in the first days after transplantation are attributed to the toxic metabolites of alkylating agents such as cyclophosphamide and busulfan and to the effect of total body irradiation on the urothelium [2]. Late-onset HC clusters around neutrophil engraftment and occurs between the second week and the sixth month; one of its principal causes is reactivation of the BK polyomavirus [3]. Reported incidence ranges from 7% to 54% in adults and from 8% to 25% in children, and high BK viruria and viraemia, myeloablative conditioning, acute graft-versus-host disease (GVHD), cytomegalovirus (CMV) viraemia, unrelated or human leukocyte antigen-mismatched donors, and cytopenias are recognised risk factors [4].

The pathogenesis of BK virus-associated haemorrhagic cystitis (BKV-HC) reflects the interaction of toxic tissue injury, viral reactivation and the inflammatory damage accompanying immune reconstitution [5]. Clinical presentation begins with irritative symptoms, and in severe cases clot retention and renal impairment develop [6]. Diagnosis rests on cystitis symptoms, Grade 2 or higher haematuria and a urinary BK viral load exceeding 7 log10 copies/mL [2]; plasma BK viral load is used to monitor the course [7].

No single treatment standard has been established [8]. Management rests on pain control, hydration, bladder irrigation, clot evacuation, transfusion support and urinary drainage; reduction in immunosuppression may support recovery of virus-specific immunity but risks exacerbating GVHD. Cidofovir has been given systemically and intravesically with variable response, and fluoroquinolones, leflunomide, brincidofovir, hyperbaric oxygen, fibrin sealant, alum and formalin have been used in different groups [6,9,10]. In refractory bleeding, arterial embolisation, urinary diversion or cystectomy are considered.

Platelet-rich plasma (PRP) contains growth factors released from platelet alpha granules that participate in cell migration, proliferation, angiogenesis and wound healing [11], and its regenerative and immunomodulatory properties provide a rationale for local application when urothelial integrity is lost. Findings in experimental cystitis models have been mixed [12,13], and the human experience is confined to a single series of ten patients [14].

One constraint has not been addressed in this literature. PRP is autologous by definition, yet allo-HSCT recipients with Grade 3–4 BKV-HC are profoundly thrombocytopenic and transfusion-dependent and cannot yield a platelet concentrate of therapeutic quality from their own blood. Any platelet-based local therapy in this population must therefore be allogeneic. Allogeneic platelet derivatives prepared by hospital transfusion services are already used topically in settings where an autologous product is unobtainable [15,16]. We therefore used an irradiated single-donor apheresis platelet concentrate, referred to throughout as allogeneic platelet concentrate (allo-PC) rather than PRP, to avoid conceptual confusion with autologous preparations. This report describes its use in patients who had exhausted every available treatment option.

## 2. Materials and Methods

### 2.1. Study Design and Ethics

This single-centre retrospective observational study reviewed the records of all patients who developed haemorrhagic cystitis after allo-HSCT at our institution between January 2018 and June 2021. The study was approved by the Non-Interventional Clinical Research Ethics Committee of Haseki Training and Research Hospital (decision no. 113-2026, 22 April 2026) and was conducted in accordance with the Declaration of Helsinki. Because the design is retrospective and the protocol specified 12 months of follow-up, ethical approval was necessarily obtained after treatment had been delivered; all clinical data had already been recorded as part of routine care, and access to the records for research purposes, data extraction and analysis were undertaken only after approval. Written informed consent was obtained from every patient, or from the parents or legal representatives of minors, before the procedure; the institutional consent form additionally recorded the patient’s agreement that clinical data arising from treatment might be used for scientific research. Reporting follows the STROBE statement [17].

### 2.2. Patient Selection

Eighty-four patients developed haemorrhagic cystitis during the study period. Sixteen had no detectable BK viruria and were not eligible. Of the 68 patients with BKV-HC, 49 responded to conservative treatment. Of the remaining 19 refractory patients, three died before the procedure could be performed. Sixteen patients underwent intravesical allo-PC, of whom one was excluded because clinical data were incomplete and one because informed consent was not documented, leaving 14 patients for analysis (Figure 1).

Inclusion required prior allogeneic HSCT, Grade 3 or Grade 4 BKV-HC, persistence of haemorrhagic cystitis despite standard conservative treatment, and detection of BK virus by polymerase chain reaction. Haemorrhagic cystitis attributable to causes other than BK virus was excluded.

### 2.3. Diagnosis and Exclusion of Alternative Causes

BKV-HC was diagnosed on the combination of clinical haemorrhagic cystitis with a urinary BK viral load above the predefined threshold of 10^7^ copies/mL. Reported BK viral loads correspond to the last measurement obtained before allo-PC administration. Urine cultures were obtained in all patients as part of standard preparation for the procedure and were negative; cystoscopy was not performed in the presence of active bacterial infection. Urinary adenovirus polymerase chain reaction was negative in all patients. CMV viraemia was recorded when present. Coagulopathy was corrected as part of routine supportive care before the intervention.

### 2.4. Treatment Before Allo-PC

All patients received hyperhydration, forced diuresis, continuous bladder irrigation through a three-way catheter, antispasmodic and analgesic therapy, and cidofovir, which is used as standard antiviral treatment for BKV-HC at our institution. In addition, immunosuppression was reduced in 10 patients, hyperbaric oxygen therapy was administered to 4, and 10 had undergone at least one previous session of cystoscopic clot evacuation with cauterisation (median 1, range 0–2). Macroscopic haematuria persisted in every patient despite these measures. Intravesical allo-PC was therefore undertaken as a salvage procedure after the available treatment options had been exhausted; the median interval between the onset of haemorrhagic cystitis and allo-PC administration was 45.5 days (IQR 38.8–63.0).

### 2.5. Preparation of the Allogeneic Platelet Concentrate

Platelets were collected by standard single-donor apheresis from a donor of the same ABO blood group or, where appropriate, an HLA-compatible donor. All donors were male, in accordance with institutional practice intended to reduce the risk of transfusion-related acute lung injury associated with HLA antibodies in previously pregnant female donors; median donor age was 30 years (range 19–53). Each unit was irradiated at 25 Gy to prevent transfusion-associated GVHD. Products were prepared and stored under standard blood-banking conditions and were collected and administered on the same day; no product was stored beyond the day of collection, and all were prepared using an identical protocol.

Ten millilitres of 10% calcium gluconate were added to the 60 mL concentrate under continuous agitation approximately 20 min before administration. Calcium gluconate was selected in preference to thrombin because thrombin preparations are heterogeneous and carry a recognised immunogenic risk that is undesirable in immunosuppressed transplant recipients, and in preference to calcium chloride because calcium deposition is less frequent in gels formed with calcium gluconate; growth factor release from calcium gluconate-activated preparations is comparable to that obtained with thrombin [18]. The 20 min interval was chosen because calcium-induced degranulation begins approximately 15 min after activation and increases progressively thereafter [19], so that administration occurs within the early release phase while the product remains fluid enough for instillation.

Platelet, leukocyte and erythrocyte concentrations were measured with an automated cell counter (Celltac, Nihon Kohden, Tokyo, Japan) after activation, in a sample drawn from the final product, and therefore reflect the preparation actually administered.

### 2.6. Procedure

Procedures were performed under general anaesthesia in the lithotomy position after a single prophylactic dose of ceftriaxone or ciprofloxacin. An 11–24 F cystoscope was selected according to age and urethral calibre, and 1.5% glycine was used for irrigation. Intravesical clots were evacuated, and bleeding areas were cauterised with a monopolar system; cauterisation was applied only to major bleeding foci and was not extensive, and the same approach was used in all patients.

Using a laparoscopic insufflator connected to the working channel, intravesical pressure was raised to 15 mmHg with carbon dioxide. This technique and pressure range follow the approach described by Kobayashi and Kamai, who used carbon dioxide insufflation at 12–15 mmHg to obtain a clear operative field and to exploit the haemostatic effect of gas pressure in a bleeding bladder [20]; gas cystoscopy has been used in urological practice since the early 1980s [21]. After 15 min, 50 mL of activated allo-PC was instilled through the working channel, and the pressure was held at 15 mmHg for a further 15 min to maintain contact with the bladder mucosa, after which a two-way urethral catheter was placed. All patients were under standard general anaesthetic monitoring throughout, including continuous end-tidal carbon dioxide, oxygen saturation, heart rate and blood pressure; no hypercapnia, acidosis or haemodynamic disturbance was recorded during insufflation.

The instilled volume was fixed at 50 mL in all patients irrespective of age or body size. Bladder capacity was not measured: cystography was deliberately avoided because the bladder wall was actively inflamed and friable in every patient and retrograde filling would have risked exacerbating haemorrhage or causing perforation. During the procedure, the 50 mL volume was instilled without resistance, and no patient showed signs of overdistension.

### 2.7. Outcome Measures

The primary outcome was resolution of macroscopic haematuria, defined as the absence of visible macroscopic haematuria on direct inspection of the urine. Microscopic haematuria was not used, as it persists for a prolonged period after cystoscopic instrumentation in catheterised patients and does not reflect clinically relevant bleeding. Secondary outcomes—post-procedural erythrocyte transfusion, time to catheter removal, readmission for haematuria and the need for salvage treatment—are reported separately rather than combined into a composite. Patients meeting all four criteria are additionally described as complete responders.

Erythrocyte transfusion followed institutional thresholds: haemoglobin below 7 g/dL in stable patients without symptoms attributable to anaemia, or below 8 g/dL in the presence of tachycardia, dyspnoea or an imminent procedure. Platelet transfusion followed separate institutional thresholds and did not contribute to any outcome definition.

### 2.8. Follow-Up

Patients remained hospitalised until macroscopic haematuria resolved and the urethral catheter was removed. Thereafter they were reviewed in person at the outpatient clinic as part of routine haematology–oncology follow-up; no telephone or remote follow-up was used. Recurrence of haematuria, recurrent haemorrhagic cystitis, urinary tract infection, late bladder complications and renal function were assessed at these visits. The median follow-up was 14.0 months (IQR 12.0–15.8). Chronic GVHD is not reported, as systematic two-year assessment was outside the scope of this study.

### 2.9. Statistical Analysis

Analyses were performed with IBM SPSS Statistics, version 26.0 (IBM Corp., Armonk, NY, USA). Given the sample size of 14 patients, the number of inferential analyses was deliberately limited, and the emphasis was placed on effect estimates with confidence intervals rather than on *p*-values. Continuous variables are presented as median (interquartile range) unless otherwise stated; proportions are reported with 95% Wilson confidence intervals. Associations between product composition and recovery times were assessed with Spearman rank correlation, reported with 95% confidence intervals obtained by Fisher z transformation. Partial rank correlations controlling for age and for body weight were computed to examine confounding. No adjustment for multiple comparisons was applied, and all analyses are exploratory and hypothesis-generating; no a priori sample size calculation was performed. All primary analyses were performed by the authors; the use of artificial intelligence is declared in Section 2.10.

### 2.10. Use of Artificial Intelligence

During the preparation of this manuscript, the authors used Claude Opus 5 (Anthropic, PBC, San Francisco, CA, USA) for the purposes of language editing, preparation of the figures, and verification of the reported statistical results against the study dataset. All primary statistical analyses were performed by the authors, and no artificial intelligence tool was used to generate, interpret or alter the clinical data. The authors have reviewed and edited the output and take full responsibility for the content of this publication.

## 3. Results

### 3.1. Patient Selection and Baseline Characteristics

Fourteen patients were analysed. The median age was 12.5 years (range 6–37) and nine (64.3%) were under 18 years. The median body weight was 35.2 kg (range 12.3–93.0). Nine patients received myeloablative conditioning with cyclophosphamide and total body irradiation and five received reduced-intensity conditioning with fludarabine and busulfan; all were therefore exposed to agents with recognised urothelial toxicity. Seven donors were related and seven unrelated, and all transplants were fully HLA-matched. Acute GVHD occurred in four patients (grade II in two, grade III in two) and CMV viraemia in three. The median neutrophil engraftment was day 16 (range 10–34) and median interval from transplantation to onset of haemorrhagic cystitis was 25.5 days (range 14–74); haemorrhagic cystitis followed neutrophil engraftment in 12 of 14 patients. The median urinary BK viral load was 3.3 × 10^8^ copies/mL (range 1.8 × 10^7^–3.2 × 10^9^), and all patients were also plasma BK-positive (median 150,000 copies/mL). Baseline characteristics are shown in Table 1 and individual patient data are shown in Supplementary Table S1a,b.

### 3.2. Administered Product and Delivered Dose

The median platelet concentration of the administered products was 986 × 10^3^/µL (range 561–1383) and median leukocyte concentration 0.3 × 10^3^/µL (range 0.1–0.9). Because a fixed 50 mL volume was instilled in every patient, the total delivered platelet dose was a median of 49.9 × 10^9^ (range 28.1–69.2). Normalised to body weight, however, the delivered dose varied more than eighteen-fold, from 0.30 to 5.62 × 10^9^ platelets/kg (median 1.11; IQR 0.72–2.67) and normalised to body surface area from 13.3 to 113.3 × 10^9^/m^2^. This heterogeneity of exposure is examined in Section 3.5.

### 3.3. Primary Outcome

Macroscopic haematuria resolved in 12 of 14 patients (85.7%, 95% CI 60.1–96.0), after a median of 5.5 days (IQR 4.0–12.5; range 3–16). The two patients in whom haematuria did not resolve were readmitted and received salvage treatment, one with fibrin sealant and one with alum irrigation.

### 3.4. Secondary Outcomes

Seven patients (50.0%, 95% CI 26.8–73.2) required no post-procedural erythrocyte transfusion, and the same seven required neither readmission nor salvage treatment; these seven therefore met all criteria previously described as complete response. The median time to catheter removal was 5.5 days (IQR 3.2–9.8), and the median postoperative hospital stay was 19.0 days (IQR 13.2–29.5). Outcomes are summarised in Table 2, and adverse events are reported separately in Table 3.

### 3.5. Product Composition, Body Weight and Recovery

Product platelet concentration correlated inversely with time to resolution of haematuria (r_s_ = −0.917, 95% CI −0.977 to −0.725), with time to catheter removal (r_s_ = −0.728, 95% CI −0.908 to −0.321) and with hospital stay (r_s_ = −0.776, 95% CI −0.925 to −0.417). Individual observations are shown in Figure 2.

Because the instilled volume was fixed, smaller patients necessarily received a larger dose per kilogram, and body weight was itself strongly associated with time to resolution (r_s_ = +0.791) and closely correlated with age (r_s_ = +0.981). Body weight, age and platelet concentration were therefore mutually entangled, and the relative contribution of each was examined by partial rank correlation (Table 4).

The association between platelet concentration and time to resolution persisted after adjustment for age (r_s_ = −0.827, *p* = 0.002) and for body weight (r_s_ = −0.783, *p* = 0.004). The association between body weight and time to resolution did not persist after adjustment for platelet concentration (r_s_ = +0.292, *p* = 0.383). Neither the interval from onset of haemorrhagic cystitis to allo-PC (r_s_ = +0.095), the plasma BK viral load (r_s_ = +0.239), the number of previous cauterisation procedures (r_s_ = −0.144), nor donor age (*p* = 0.70) was associated with outcome.

## 4. Discussion

In this single-centre series of 14 patients with Grade 3–4 BKV-HC, macroscopic haematuria resolved in 12 (85.7%) after intravesical administration of an allogeneic platelet concentrate, at a median of 5.5 days. The distinguishing feature of the cohort is not the response rate but the clinical situation in which the intervention was used: every patient had already failed hyperhydration, forced diuresis, continuous bladder irrigation and cidofovir; 10 had undergone at least one previous cystoscopic clot evacuation with cauterisation; 10 had immunosuppression reduced; and 4 had received hyperbaric oxygen. Haematuria had persisted for a median of 45.5 days before the procedure. These patients had, in other words, exhausted the treatment options available to us.

This history bears directly on two alternative explanations for the observed improvement. The first is that recovery followed the cystoscopic clot evacuation and cauterisation performed immediately before instillation rather than the platelet product. This remains possible and cannot be excluded, and the procedure must be understood as a combined intervention. It is nonetheless relevant that the same cystoscopic procedure had already been performed at least once in 10 of the 14 patients without resolution of bleeding, and that cauterisation was deliberately limited to major bleeding foci rather than applied extensively. The second is spontaneous resolution during the natural course of BKV-HC. Spontaneous improvement does occur, and 49 of the 68 patients with BKV-HC treated during the study period recovered with conservative management alone and never required this procedure. The 14 patients reported here are precisely those in whom that did not happen over a median of six weeks of continuous treatment.

The product used here differs fundamentally from PRP as conventionally defined. PRP is autologous, whereas our patients were thrombocytopenic transplant recipients from whom an autologous concentrate of therapeutic quality could not be obtained. The product is therefore designated an allogeneic platelet concentrate throughout. The distinction is not semantic: donor-derived variation in platelet yield becomes an independent source of variability in the delivered dose, which is what the association between product platelet concentration and outcome reflects. It also means these findings cannot be extrapolated to the autologous PRP literature, and that the applicable safety framework is that of transfusion medicine.

The relationship between product composition and recovery requires careful interpretation. Because a fixed 50 mL volume was instilled irrespective of patient size, the delivered dose varied more than eighteen-fold per kilogram, and body weight was itself associated with time to resolution. Age, body weight and platelet concentration were mutually correlated, and a single small cohort cannot fully separate them. The partial correlation analysis is nevertheless informative: the association between platelet concentration and time to resolution persisted after adjustment for both age and body weight, whereas the association between body weight and time to resolution disappeared after adjustment for platelet concentration. The most parsimonious reading is that the apparent effect of body size operated through the platelet dose delivered rather than independently of it. This does not establish causation, and we do not claim that platelet concentration determines recovery; it does indicate that the association is not simply a restatement of patient age or size.

The mechanistic interpretation remains hypothetical. We measured platelet, leukocyte and erythrocyte concentrations but not growth factors, cytokines, chemokines, platelet activation markers or any tissue-level healing response. The proposition that a higher platelet load delivers greater concentrations of growth factors and a denser fibrin matrix to the damaged urothelium is therefore a hypothesis rather than a demonstrated mechanism, and platelet count is at best a surrogate for biological activity [11]. Because the product is prepared at the point of care, however, this surrogate is modifiable, and its association with outcome argues for standardised preparation and routine reporting of product characteristics in future studies.

Human data on intravesical platelet preparations remain very limited. In the preliminary series of Masieri et al., which evaluated 10 patients with refractory Grade 3–4 BKV-HC, six achieved complete and three partial responses, with a median time to catheter removal of 6 days and a hospital stay of 35 days [14]. Our median time to catheter removal of 5.5 days is similar. These comparisons are offered as context only: patient ages, underlying diseases, supportive care, prior treatment, discharge criteria and outcome definitions differ, and no inference of superiority is intended. The same qualification applies to the comparison with hyperbaric oxygen [9,10], particularly since four of our patients had already failed that treatment.

Several limitations must be acknowledged. First and most importantly, there was no control group, and the improvement observed cannot be attributed to the platelet concentrate with confidence. Second, the cohort was predominantly paediatric—nine of fourteen patients were under 18 years—which limits extrapolation to adult transplant recipients, and age was associated with both product characteristics and outcome. Third, a fixed volume was administered irrespective of body size; future studies should define dosing by weight, body surface area or bladder capacity. Fourth, bladder capacity was not measured, for the reasons given in the Methods. Fifth, the number of patients is small, and although the number of inferential analyses was deliberately limited, all estimates are unstable and should be read together with their confidence intervals rather than their *p*-values. Sixth, product characterisation was limited to cell concentrations. Seventh, chronic GVHD and long-term urological outcomes beyond a median of 14 months were not assessed. Finally, the findings derive from a single centre.

## 5. Conclusions

In 14 patients with Grade 3–4 BK virus-associated haemorrhagic cystitis who had failed conservative treatment, cidofovir and, in most cases, previous cystoscopic cauterisation, immunosuppression reduction or hyperbaric oxygen, intravesical administration of an irradiated single-donor allogeneic platelet concentrate was followed by resolution of macroscopic haematuria in 12 patients at a median of 5.5 days, without procedure-related complications exceeding Clavien–Dindo Grade I. The platelet content of the administered product was associated with time to resolution, and this association was not explained by patient age or body weight. Because the study is uncontrolled and the procedure combined cystoscopic clot evacuation with instillation, these findings are hypothesis-generating and require confirmation in prospective controlled studies using standardised, weight-adjusted dosing.

## Figures and Tables

**Figure 1:**
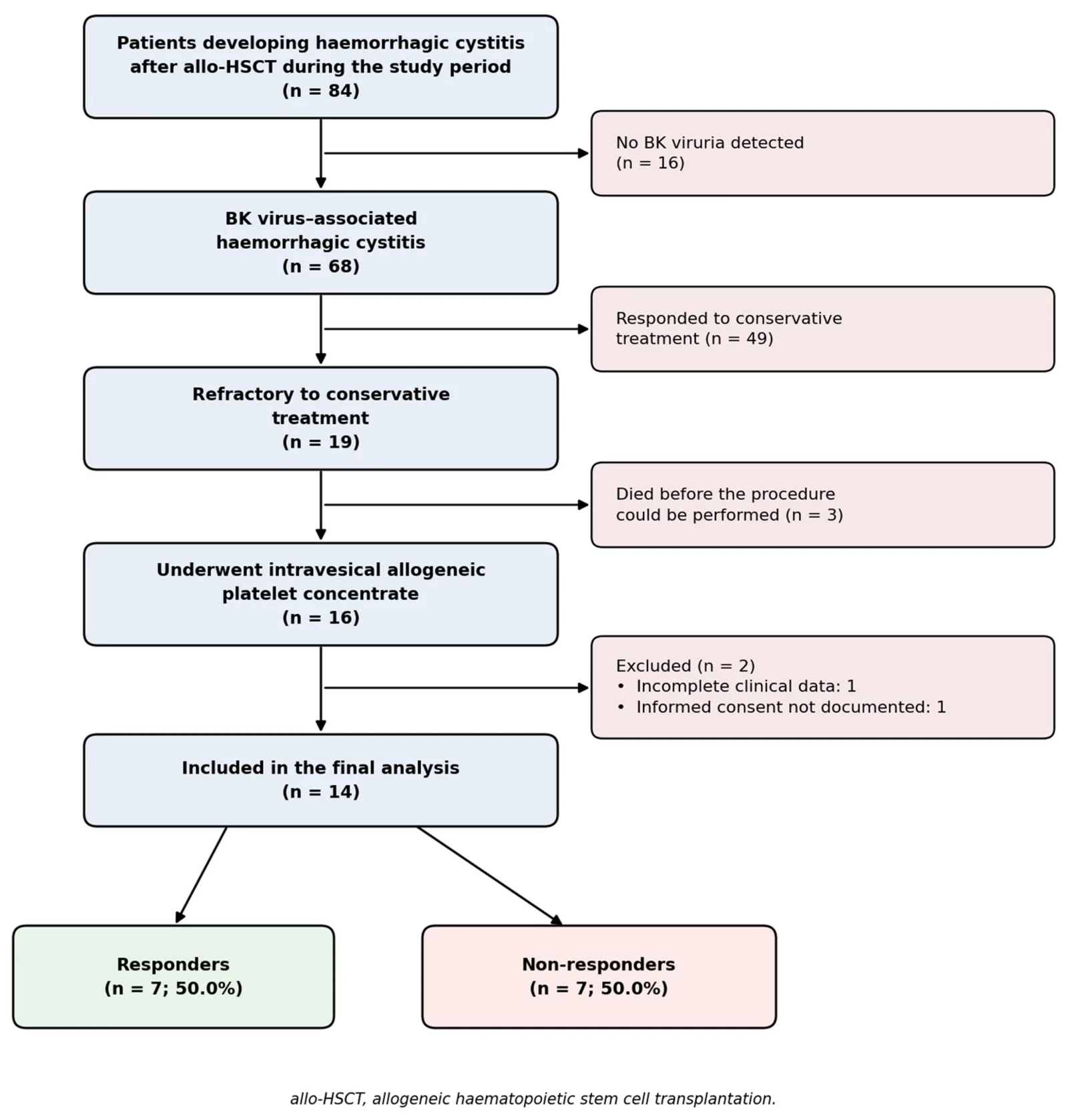
Flow of patients through the study.

**Figure 2:**
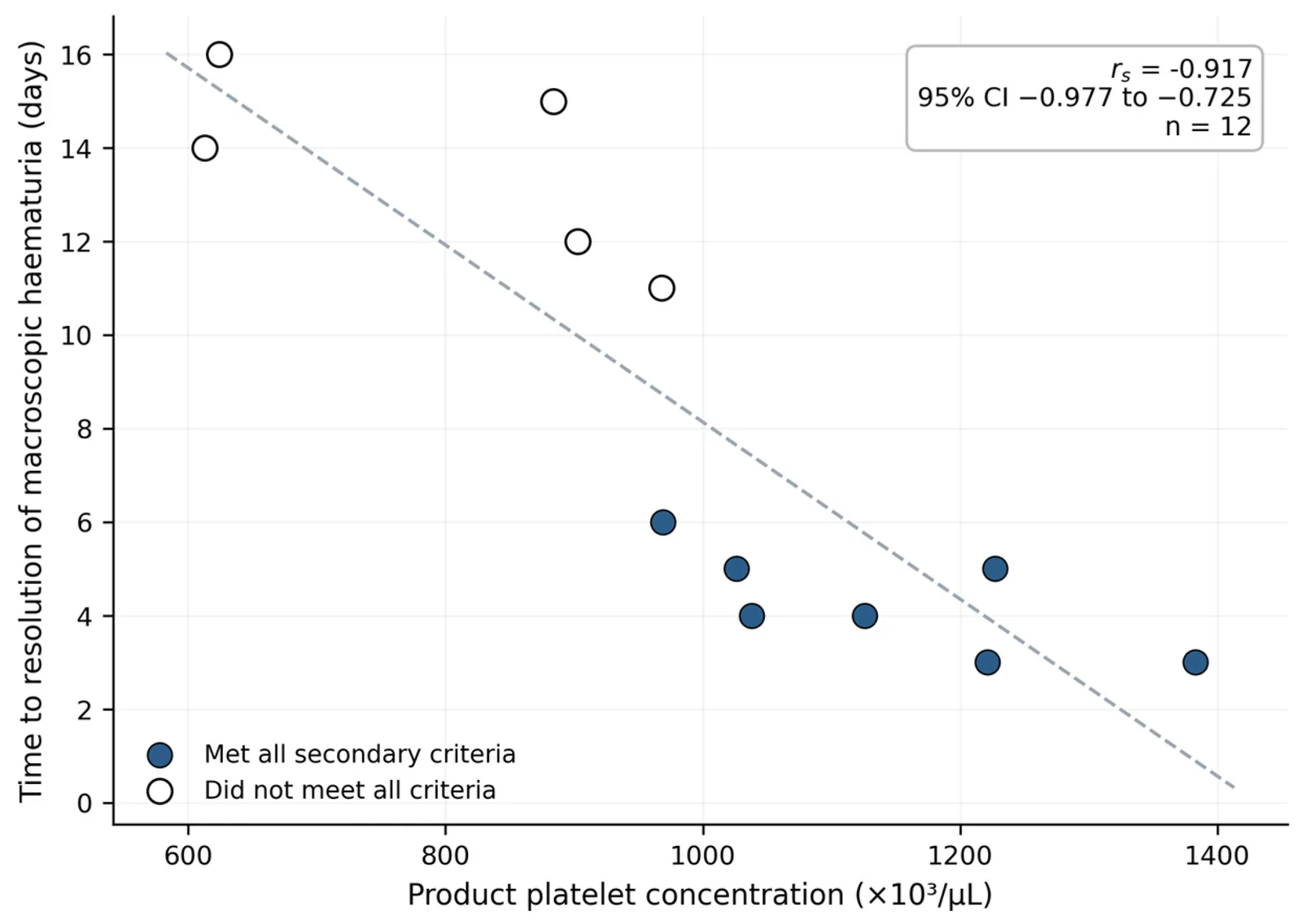
Product platelet concentration and time to resolution of macroscopic haematuria in the 12 patients whose haematuria resolved. Individual observations are shown; the dashed line is a least-squares fit for orientation only, and the reported coefficient is a Spearman rank correlation. Given the small number of observations, this association should be regarded as exploratory.

**Table 1:** Baseline characteristics (*n* = 14).

Variable	Value
Age (years), median (range)	12.5 (6–37)
Age < 18 years, *n* (%)	9 (64.3)
Male sex, *n* (%)	9 (64.3)
Body weight (kg), median (range)	35.2 (12.3–93.0)
§Diagnosis, *n* (%)	
Acute myeloid leukaemia	5 (35.7)
Acute lymphoblastic leukaemia	5 (35.7)
Severe aplastic anaemia	2 (14.3)
Myelodysplastic syndrome	2 (14.3)
§Conditioning, *n* (%)	
Myeloablative (Cy + TBI)	9 (64.3)
Reduced-intensity (Flu + Bu)	5 (35.7)
Related donor, *n* (%)	7 (50.0)
Full HLA match, *n* (%)	14 (100)
Acute GVHD, *n* (%)	4 (28.6)
CMV viraemia, *n* (%)	3 (21.4)
Neutrophil engraftment (day), median (range)	16 (10–34)
Transplant to HC onset (days), median (range)	25.5 (14–74)
HC onset to allo-PC (days), median (IQR)	45.5 (38.8–63.0)
Urinary BK load (copies/mL), median	3.3 × 10^8^
Plasma BK load (copies/mL), median (range)	150,000 (7200–8.0 × 10^6^)
§HC grade, *n* (%)	
Grade 3	6 (42.9)
Grade 4	8 (57.1)
§Failed prior treatment, *n* (%)	
Hyperhydration, irrigation, cidofovir	14 (100)
Previous cystoscopic cauterisation	10 (71.4)
Immunosuppression reduction	10 (71.4)
Hyperbaric oxygen	4 (28.6)

Cy, cyclophosphamide; TBI, total body irradiation; Flu, fludarabine; Bu, busulfan; GVHD, graft-versus-host disease; CMV, cytomegalovirus; HC, haemorrhagic cystitis; allo-PC, allogeneic platelet concentrate.

**Table 2:** Clinical outcomes (*n* = 14).

Outcome	Value
Primary outcome	
Resolution of macroscopic haematuria, *n* (%)	12 (85.7); 95% CI 60.1–96.0
Time to resolution (days), median (IQR)	5.5 (4.0–12.5)
Secondary outcomes	
No post-procedural erythrocyte transfusion, *n* (%)	7 (50.0); 95% CI 26.8–73.2
Time to catheter removal (days), median (IQR)	5.5 (3.2–9.8)
No readmission for haematuria, *n* (%)	12 (85.7)
No salvage treatment required, *n* (%)	12 (85.7)
Postoperative hospital stay (days), median (IQR)	19.0 (13.2–29.5)
Follow-up (months), median (IQR)	14.0 (12.0–15.8)
Met all four criteria (previously ‘complete response’), *n* (%)	7 (50.0)

CI, confidence interval; IQR, interquartile range. Secondary outcomes are reported separately rather than combined into a composite endpoint.

**Table 3:** Procedure-related adverse events (*n* = 14).

Adverse Event	*n* (%)	Clavien–Dindo
Bladder spasm	5 (35.7)	I
Catheter obstruction by clot	3 (21.4)	I
Urinary tract infection	0 (0)	—
Other infection	0 (0)	—
Bladder perforation	0 (0)	—
Anaesthesia-related event	0 (0)	—
Transfusion reaction	0 (0)	—
Carbon dioxide-related event	0 (0)	—
Any event above Grade I	0 (0)	—

Adverse events are reported here independently of efficacy outcomes. Erythrocyte transfusion is reported in Table 2 as a clinical outcome of the underlying disease and is not counted here as a procedural complication, since it reflected ongoing bleeding rather than a complication of the intervention. No event required surgical, endoscopic or radiological intervention.

**Table 4:** Partial rank correlations with time to resolution of macroscopic haematuria (*n* = 12).

Variable	Unadjusted r_s_	*p*	Adjusted r_s_	*p*
Platelet concentration, adjusted for age	−0.917	<0.001	−0.827	0.002
Platelet concentration, adjusted for body weight	−0.917	<0.001	−0.783	0.004
Body weight, adjusted for platelet concentration	+0.791	0.002	+0.292	0.383
Dose per kilogram (unadjusted)	−0.805	0.002	—	—
Dose per body surface area (unadjusted)	−0.826	0.001	—	—

Partial Spearman rank correlation. The association between product platelet concentration and time to resolution persisted after adjustment for both age and body weight, whereas the association with body weight did not persist after adjustment for platelet concentration.

## Data Availability

Individual patient data are provided in Supplementary Table S1a,b. Further data are available from the corresponding author on reasonable request.

## Supplementary Materials

The following supporting information can be downloaded at: https://www.mdpi.com/article/10.3390/jcm15197407/s1. Table S1a. Patient and transplant characteristics (*n* = 14); Table S1b. Prior treatment, administered product and outcomes (*n* = 14).
