# Supplementary material for: Intravesical Allogeneic Platelet Concentrate for Refractory BK Virus-Associated Haemorrhagic Cystitis After Allogeneic Haematopoietic Stem Cell Transplantation
Source: J Clin Med. 2026 Sep 24;15(19):7407. doi: 10.3390/jcm15197407 (PMC13666128; doi:10.3390/jcm15197407)
Supplement: Supplementary file 1 [file jcm-15-07407-s001.zip › jcm-4562815-supplementary.pdf]

Supplementary Material

Individual patient data are presented in two tables rather than one. Supplementary Table S1a describes the patients and their transplants; Supplementary Table S1b describes the treatment they had already received, the product administered and the outcomes. The two tables share the same patient identifiers and can be read together. They were separated because a single table containing all variables requested at review could not be presented at a legible font size.

Supplementary Table S1a. Patient and transplant characteristics (n = 14).

Pt	Age	Sex	Wt (kg)	Diagnosis	Cond.	Donor	HLA	Eng. (d)	aGVHD	CMV	Tx→HC (d)	Urine BK (copies/mL)	Plasma BK (copies/mL)	HC grade
P01	26	M	64.4	AML	MAC	Related	Full	14	–	+	31	3.2×109	8,000,000	3
P02	37	M	72.4	ALL	MAC	Unrelated	Full	25	–	–	42	2.7×108	900,000	4
P03	37	M	92.5	SAA	RIC	Unrelated	Full	11	II	–	16	3.2×107	1,200,000	4
P04	23	M	83.9	ALL	MAC	Unrelated	Full	17	–	–	20	1.1×109	850,000	4
P05	11	F	28.7	AML	RIC	Unrelated	Full	34	–	–	28	1.5×109	600,000	3
P06	8	F	21.3	ALL	MAC	Related	Full	15	II	–	23	4.5×108	350,000	4
P07	10	M	22.7	MDS	MAC	Related	Full	10	–	+	18	1.6×109	7,200	4
P08	19	M	93.0	ALL	MAC	Unrelated	Full	18	III	–	42	3.0×108	180,000	3
P09	6	M	12.3	AML	MAC	Related	Full	13	–	–	19	3.6×108	120,000	3
P10	6	M	15.2	AML	RIC	Unrelated	Full	16	III	–	14	2.0×108	85,000	3
P11	8	F	20.7	MDS	RIC	Related	Full	20	–	+	67	1.8×107	60,000	4
P12	10	F	21.9	SAA	MAC	Related	Full	32	–	–	34	3.0×107	15,000	3
P13	14	M	41.8	AML	MAC	Related	Full	14	–	–	21	1.9×107	25,000	4
P14	14	F	45.5	ALL	RIC	Unrelated	Full	16	–	–	74	7.1×108	8,500	4

Pt, patient; Wt, body weight; Cond., conditioning regimen (MAC, myeloablative — cyclophosphamide and total body irradiation; RIC, reduced-intensity — fludarabine and busulfan); Donor, donor relationship; HLA, degree of HLA match; Eng., day of neutrophil engraftment after transplantation; aGVHD, acute graft-versus-host disease grade; CMV, cytomegalovirus viraemia; Tx→HC, interval from transplantation to onset of haemorrhagic cystitis; HC grade, haemorrhagic cystitis grade. BK viral loads correspond to the last measurement obtained before the procedure.

Supplementary Table S1b. Prior treatment, administered product and outcomes (n = 14).

Pt	HC→PC (d)	Prior caut. (n)	HBO	IS red.	PLT ($\times10^3/\mu L$)	WBC ($\times10^3/\mu L$)	Dose ($\times10^9$)	Dose/kg ($\times10^9$)	Resolution (d)	Catheter (d)	RBC tx	Salvage	Stay (d)	F-up (mo)	Outcome
P01	64	2	+	+	1044	0.9	52.2	0.81	NR	21	+	Fibrin sealant	52	10	NR
P02	46	1	+	+	1026	0.3	51.3	0.71	5	4	–	–	15	17	CR
P03	66	1	+	–	624	0.5	31.2	0.34	16	12	+	–	35	15	PR
P04	45	1	–	+	884	0.3	44.2	0.53	15	10	+	–	30	13	PR
P05	38	1	–	+	613	0.2	30.7	1.07	14	9	+	–	28	12	PR
P06	71	2	–	–	1221	0.3	61.1	2.87	3	2	–	–	11	18	CR
P07	33	1	–	+	903	0.9	45.2	1.99	12	8	+	–	25	16	PR
P08	69	0	+	–	561	0.7	28.1	0.30	NR	18	+	Alum	45	9	NR
P09	37	1	–	+	1383	0.2	69.2	5.62	3	2	–	–	10	15	CR
P10	41	1	–	–	1126	0.4	56.3	3.70	4	3	–	–	12	14	CR
P11	53	1	–	+	1227	0.3	61.4	2.96	5	4	–	–	13	12	CR
P12	31	0	–	+	1038	0.1	51.9	2.37	4	3	–	–	14	14	CR
P13	45	0	–	+	968	0.6	48.4	1.16	11	7	+	–	22	11	PR
P14	60	0	–	+	969	0.2	48.5	1.06	6	4	–	–	16	20	CR

Pt, patient; HC→PC, interval from onset of haemorrhagic cystitis to administration of the allogeneic platelet concentrate; Prior caut., number of previous cystoscopic clot evacuation and cauterisation procedures performed before the index procedure; HBO, hyperbaric oxygen therapy given before the index procedure; IS red., reduction of immunosuppression; PLT/WBC, platelet and leukocyte concentration of the administered product, measured after activation; Dose, total platelet dose delivered in the 50 mL instilled volume; Resolution, time to resolution of macroscopic haematuria (NR, not resolved); Catheter, time to catheter removal; RBC tx, post-procedural erythrocyte transfusion; Salvage, salvage treatment required after the procedure; Stay, postoperative hospital stay; F-up, follow-up duration; Outcome: CR, haematuria resolved and all secondary criteria met; PR, haematuria resolved but one or more secondary criteria not met; NR, haematuria did not resolve. All 14 patients had received hyperhydration, forced diuresis, continuous bladder irrigation and cidofovir before the procedure without resolution of bleeding.
